# Hand-assisted laparoscopic suture rectopexy for complete rectal prolapse complicated by a solitary ulcer and obstructed defecation: a case report and review of the literature

**DOI:** 10.1186/1752-1947-7-133

**Published:** 2013-05-30

**Authors:** Narimantas Evaldas Samalavičius, Edvinas Kildušis

**Affiliations:** 1 Center of Oncosurgery, Institute of Oncology, Vilnius University, Santariskiu str. 1, Vilnius, LT-08660, Lithuania

**Keywords:** Rectal prolapse, Solitary rectal ulcer syndrome, Hand-assisted laparoscopy, Suture rectopexy.

## Abstract

**Introduction:**

Solitary rectal ulcer syndrome is a condition in which an ulcer occurs in the rectum. There is evidence that solitary rectal ulcer syndrome is associated with rectal prolapse either overt or occult and that stopping complete rectal prolapse may lead to rapid healing of the solitary rectal ulcer. A huge variety of operative techniques have been described in the literature to correct this condition. We present the case of a patient who underwent hand-assisted laparoscopic suture rectopexy for complete rectal prolapse complicated by a solitary ulcer and obstructed defecation.

**Case presentation:**

A 32-year-old Caucasian woman presented to our institute complaining of having had difficulty with her bowel movements, a rectal prolapse and pain in the anal area for one and a half years. She was checked in hospital for suspected rectal carcinoma, however, the examination revealed rectal ulceration. A diagnosis of complete rectal prolapse complicated by a solitary ulcer and obstructed defecation was established. The symptoms persisted so a hand-assisted laparoscopic suture rectopexy was performed. After six months of follow-up, her bowel movements had improved, she was experiencing no pain and the rectal ulcer had healed.

**Conclusion:**

A hand-assisted laparoscopic suture rectopexy is a feasible and safe surgical treatment of rectal prolapse with solitary rectal ulcer syndrome, providing complete recovery for patients with solitary rectal ulcer syndrome.

## Introduction

Complete rectal prolapse (procidentia) is the protrusion of the entire thickness of the rectal wall through the anal sphincter complex. Patients with rectal prolapse suffer from anal incontinence (50 to 75 percent), constipation (30 to 50 percent), mucus or blood discharge from the protruding tissue (25 percent) and pain during bowel movements [1-3]. There is evidence that solitary rectal ulcer syndrome (SRUS) is associated with rectal prolapse either overt or occult. SRUS is a chronic disease in which a benign ulceration area develops in the rectum [4]. This case confirms that stopping complete rectal prolapse may lead to rapid healing of the solitary rectal ulcer. Historically the standard treatment for complete rectal prolapse consists of surgery with a transabdominal or perineal approach. Abdominal rectopexy gives low recurrence rates and functional improvement in the majority of cases. However, rectal prolapse is a disease primarily affecting older people, so various perineal approaches are also used [5]. A possible alternative is laparoscopic rectopexy [6] or hand-assisted laparoscopic rectopexy [7,8]. It represents the latest development in the evolution of surgical treatment of rectal prolapse and it is one of the main surgical techniques for the treatment of SRUS, providing complete recovery for patients with SRUS [9,10]. This method gives the good functional outcome of the abdominal procedure and the benefits of minimally invasive surgery too [11-13].

## Case presentation

**Figure 1:**
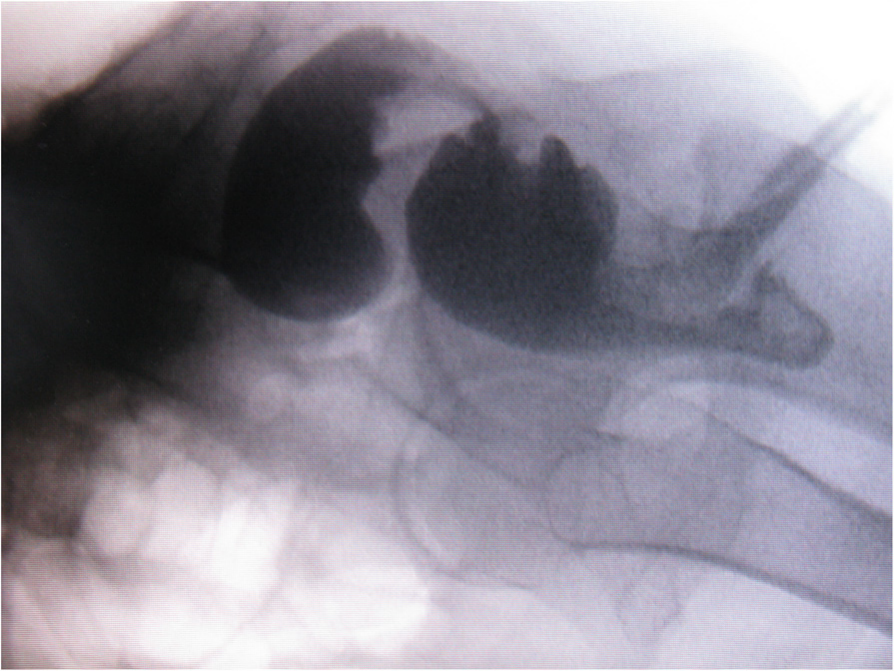
Proctography.

**Figure 2:**
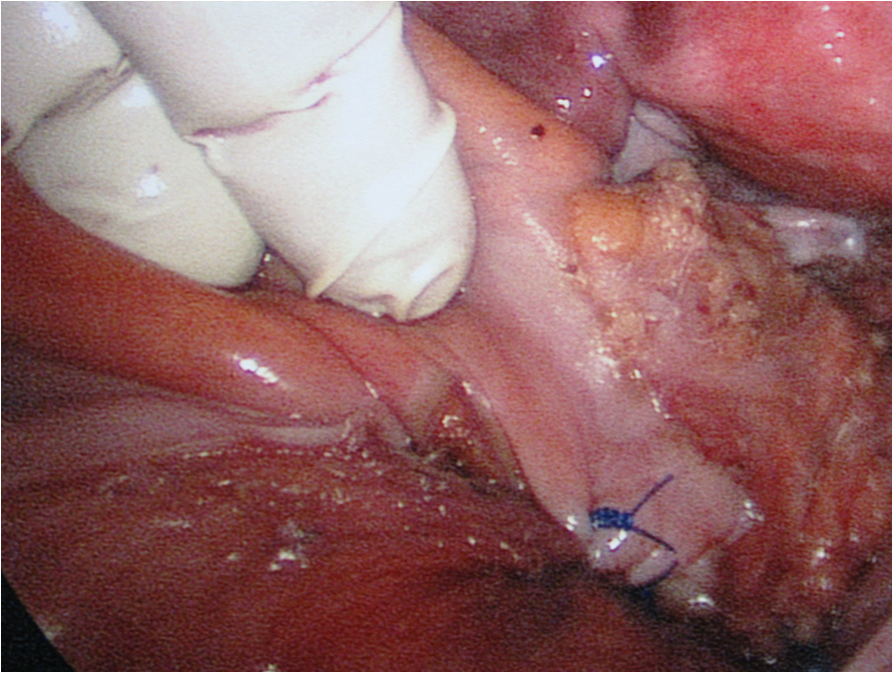
Fixation of the rectum to the presacral fascia.

We report the case of a 32-year-old Caucasian woman who was complaining of having difficulty with her bowel habits, a rectal prolapse and pain in the anal area. She had been experiencing difficulty with her bowel movements for one and a half years; sometimes, the patient observed blood in her feces and the rectum fell out during straining. Because of the soreness and difficulty in defecation, she was examined in our hospital for suspected rectal carcinoma. Sigmoidoscopy was performed and ulceration was observed in the middle third of the rectum, followed by a biopsy. The result of the pathistological examination was an ulcer. The patient was discharged from the hospital for out-patient follow-up without a confirmed diagnosis of tumor. The symptoms persisted. During a second hospitalization, proctoscopy revealed a stricture of the middle part of the rectum and rectocele (Figure 1). During sigmoidoscopy, 9cm from the anus, rectum-narrowing ulceration was found, so a biopsy of the impaired region was repeated and the pathistological result was the same: an ulcer. A diagnosis of third-degree complete rectal prolapse complicated by a solitary ulcer and obstructed defecation was established and a hand-assisted laparoscopic suture rectopexy was performed (Figure 2). After six months of follow-up, her bowel movements had improved, she was experiencing no pain and the rectal ulcer had healed.

## Discussion

Rectal prolapse or procidentia is a disabling problem for patients and it is not an uncommon condition [14]. The disease was described in the Egyptian Ebers Papyrus in 1500 BC, but despite this etiology and pathophysiology, and like the surgical management of the disease, it remains highly controversial until now [15].

Some authors think that rectal prolapse and SRUS are heterogeneous in their etiology [16], but there is evidence that the two disorders are associated with each other [4]. SRUS was first described in 1829 and its clinicopathological features were reported in 1969 [9].

Hippocrates described the procedure of shaking the patient while hanging him by the heels to reduce the prolapse [15]. Since then, many surgical techniques to treat full-thickness rectal prolapse have been reported. A wide variety of surgical procedures (rectopexy, anterior resection or a combination of both, perineal rectosigmoidectomy, mucosal sleeve resection or anal encirclement) and current results show that doubt still exists about the ‘ideal choice’ of ‘optimal’ operation for complete rectal prolapse [15]. These facts have influence on surgeons’ operation selection and they tend to use methods with which they are familiar. Despite that, the management of rectal prolapse is usually surgical [15]. The aim of surgery in rectal prolapse is first, to control the prolapse and second, the correction of the impaired anorectal physiology [1].

Surgical approaches are classified into transabdominal and perineal. Perineal procedures were first used in the late 19th century and, in 1939, Pemberton and Stalker described the first abdominal suspension and fixation of the rectum for the treatment of rectal prolapse [17]. According to the literature, it is accepted that an abdominal approach (transabdominal procedures such as mesh or suture rectopexy and resection-suture rectopexy) has an acceptably lesser recurrence rate and better function than the perineal approach, but the latter has a lower complication rate, minimal morbidity and a shorter length of stay in hospital. Although it is argued that a perineal approach is reserved for older or sicker patients who belong to a high surgical risk category [6,15].

High recurrence rates for primary and repeat Delorme’s operations are present after surgical management, but this method has an advantage of being less traumatic to the patients and is well tolerated by frail, older or medically unfit patients. However, it should also be considered as a primary procedure for young women who wish to have a family, those with constipation or patients with a short prolapse and a normal pelvic floor, and in men in whom the risk of impotence following an abdominal procedure is unacceptably high [18].

Other surgical modality resection rectopexy is reserved for patients who have a history of severe constipation, but here there is the problem associated with anastomosis [15]. In cases of recurrence, resectional procedures may result in an ischemic segment between two anastomoses, unless the surgeon can resect a previous anastomosis in the repeat procedure [12,15].

The feasibility, safety and effectiveness of laparoscopic surgery in the management of complete rectal prolapse have been demonstrated in several recent reports and it has the same clinical and functional results and with the same risk of operative complications as open rectopexy, but with less postoperative pain, earlier return of gastrointestinal function, better cosmesis, shorter postoperative stay and lower costs [13,19].

Another problem already mentioned above is that SRUS is associated with rectal prolapse [4], fortunately, Sitzler *et al*. in 1998 performed a retrospective study and found that anti-prolapse operations (mainly rectopexy) result in a satisfactory long-term outcome in about 55 to 60 percent of patients having surgery for SRUS [10]. Furthermore, Halligan *et al*. in their studies reported that 94 percent of patients with SRUS had complete remission of rectal prolapse after rectopexy [20].

In agreement with published data that suggest stopping complete rectal prolapse may lead to rapid healing of the solitary rectal ulcer [4], we decided to perform laparoscopic suture rectopexy as the best method of management of rectal prolapse associated with SRUS and succeeded with a very good clinical outcome at the six-month follow-up.

Despite the efforts of surgical management of complete rectal prolapse, overall recurrence rate is greater than 15 percent [21]. Causes for recurrent complete rectal prolapse were most often attributable to problems with the mesh, because there is always a risk of infection with synthetic material used in operations, especially with resection, but it may be due to an infected pelvic hematoma in cases without resection [22]. Preoperative incontinence, constipation and rectal ulcer were largely unchanged by recurrent complete rectal prolapse operations [23].

## Conclusion

Hand-assisted laparoscopic suture rectopexy is a feasible and safe surgical treatment of rectal prolapse with SRUS, providing complete recovery for operated patients with SRUS and offers benefits such as less postoperative pain, earlier return of gastrointestinal function, better cosmesis, shorter postoperative stay and lower costs.

## Consent

Written informed consent was obtained from the patient for publication of this case report and accompanying images. A copy of the written consent is available for review by the Editor-in-Chief of this journal.

## Competing interests

The authors declare that they have no competing interests.

## Authors’ contributions

NES and EK examined and treated the patient, analyzed and interpreted the patient’s data. All authors read and approved the manuscript.
